# Effect of waiting time on patient satisfaction in outpatient: An empirical investigation

**DOI:** 10.1097/MD.0000000000035184

**Published:** 2023-10-06

**Authors:** Hui Zhang, Weimin Ma, Shufen Zhou, Jingjing Zhu, Li Wang, Kaixin Gong

**Affiliations:** a School of Economics and Management, Tongji University, Shanghai, China; b School of Management, Shanghai University of Engineering Science, Shanghai, China; c Scientific Research Department, The First Affiliated Hospital of Zhejiang Chinese Medical University (Zhejiang Provincial Hospital of Traditional Chinese Medicine), Hangzhou, China; d Eye Hospital, Wenzhou Medical University at Hangzhou, Zhejiang Eye Hospital at Hangzhou, Hangzhou, China.

**Keywords:** objective waiting time, satisfaction score, subjective waiting time

## Abstract

This study aimed to identify the effect of waiting time on patient satisfaction and the relationship between different types of waiting time. The questionnaire contained 2 parts. The first part included questions about expected waiting time (EWT), reasonable waiting time, tolerance waiting time, and basic personal information. The second part included perceived waiting time (PWT) and satisfaction evaluation. The actual waiting time (AWT) was recorded by the worker. Linear regression was used to analyze the influence of waiting time on satisfaction. Before data collection, this study was approved by the hospital’s health ethics committee. In total, 323 questionnaires were collected, of which 292 (90.4%) were valid. The EWT, tolerance waiting time, rational waiting time, and PWT had a significant effect on patient satisfaction (*P* = .006, *P* = .043, *P* = .009, *P* = .000), whereas AWT had no significant effect on satisfaction (*P* = .365). The difference between the EWT and AWT had a significant effect on satisfaction (*P* = .000), while the difference between the PWT and AWT had a significant effect on satisfaction (*P* = .000). Age, educational background, gender, appointment, and hospital visit experience had no significant effect on patient satisfaction (*P* = .105, *P* = .443, *P* = .260, *P* = .352, *P* = .461, respectively). Patient satisfaction with waiting time was not directly affected by AWT, but by subjective waiting times. Furthermore, objective waiting time affects patient satisfaction through the subjective waiting time. Therefore, hospital managers can improve service quality by focusing on adjusting a patient’s subjective waiting time while reducing the objective waiting time.

## 1. Introduction

Patient satisfaction is considered an indicator of medical quality.^[1]^ To improve patient satisfaction, many efforts have focused on reducing the actual waiting time (AWT).^[2–4]^ The methods for AWT reduction include appointment,^[5,6]^ scheduling,^[7]^ and the use of artificial intelligence to arrange images in advance.^[8,9]^ These measures have reduced the AWT of outpatients,^[10]^ but patients are still dissatisfied with the waiting time.^[11]^ It shows that the AWT does not have a significant effect on patient satisfaction.^[12]^ Then, in the category of time factors, in addition to AWT, the effect of other time factors on patient satisfaction needs to be determined.

Satisfaction is the subjective evaluation of patients and is affected by subjective factors. Some scholars have noticed that patient satisfaction is affected by AWT as well as subjective factors such as expected waiting time (EWT)^[13,14]^ and perceived waiting time (PWT).^[15,16]^

Irrational individuals make decisions or evaluations that are not completely based on the current objective situation, but are also based on reference points.^[17,18]^ In the value function model of prospect theory, if the actual situation is better than the reference point, it is regarded as obtained, and the individual tends to give a positive evaluation; by contrast, if the actual situation is worse than the reference point, it is regarded as a loss, and the individual tends to give a negative evaluation.^[19]^ EWT is the reference point in the evaluation of waiting time. If the AWT is far greater than the EWT, then patients tend to give a negative evaluation; otherwise, they tend to give a positive evaluation of the waiting time.^[13]^ PWT is another important subjective waiting time, which has attracted attention in the field of service^[20]^ and affects the evaluation of medical service quality.^[21]^

Some people who waited for an hour felt as if they had waited for a century, while others also waited for an hour felt like they only waited for a few minutes. People’s subjective estimates of AWT are sometimes longer than perceived and sometimes shorter than it;^[22]^ to some extent, this condition is related to their tolerance waiting time (TWT). If patients have other arrangements on the day of treatment, the waiting time will have a TWT. When the AWT exceeds the patient’s TWT, patients become increasingly impatient, leading to obvious physical and mental discomfort.^[23]^ The reasonable waiting time (RWT) is the evaluation of the rationality of the patient’s waiting time, and it does exist in the waiting process.

This study aimed to investigate the impact of waiting time on patient satisfaction, with a specific focus on enhancing our understanding of the relationship between outpatient waiting times and patient satisfaction. Waiting time was measured using both objective and subjective factors, including AWT, estimated wait time (EWT), PWT, RWT and TWT. This study examined the effect of the interaction between objective and subjective waiting times on patient satisfaction. Finally, the analysis investigated how basic personal factors, such as gender and age, influence waiting times.

The innovation of this study includes 3 aspects. First, regarding factors of satisfaction, in addition to the AWT, the EWT, PWT, TWT, and RWT were included in the scope of the study according to the actual situation, making this study more consistent with the actual situation when discussing the factors influencing patient satisfaction. Second, this study focused on the combined effects of various time factors on satisfaction. Finally, a smart questionnaire implementation process is designed to obtain the patient’s subjective waiting time.

## 2. Methods

### 2.1. Questionnaire design

We investigated the time factors that led to patient dissatisfaction. A 2-part questionnaire contained 2 parts was designed. In the first part of the questionnaire, patients EWT, RWT, and TWT were asked directly, along with basic personal information including gender, education, and so on. In the second part of the questionnaire, the patients’ PWT and satisfaction with waiting time were assessed. The expression of patient satisfaction is based on a score of 0 to 100, with “0” indicating very dissatisfied and “100” indicating very satisfied. The higher the score, the higher is the level of satisfaction. The staff recorded the AWT of the patient, which was the difference between the time of entering the clinic and the time of registration.

### 2.2. Subjects and settings

The study participants were patients who visited the hospital for treatment of various visual problems. The reason for choosing this type of patient was to exclude the impact of objective factors such as physical pain and disease severity on the satisfaction evaluation results. The formula for sample size was expressed as follows: $n=\frac{Z^{2}P\left(1-P\right)}{E^{2}}$, where *n* is the minimum sample size, Z is the normal standard deviation at a 95% confidence level (1.96), and P is the prevalence of the factor in the study, which was determined to be 80% based on previous studies.^[24]^ In the optometry clinic in August 2022, outpatient patients who visited 3 doctors offices on Saturday mornings were selected. The 3 doctors we selected were all ordinary specialist doctors, and the reason for not choosing experts was that it was difficult to register with the expert number. The chance for patients to register with the expert number will offset the unpleasant waiting experience.

### 2.3. Questionnaire implementation

Considering that the actual waiting time and perceived waiting time only occur when the patient is called into the doctor’s office, but at that time, if the patient fills out the entire questionnaire, it will interfere with the normal medical order due to the long filling time. Therefore, the implementation of the questionnaire included 3 steps: the first part of the questionnaire was completed when patients entered the waiting room, and the second part, which contained only 2 questions, was completed when patients were called to enter the consulting room. The actual waiting time in the third part was recorded by the staff.

In the first stage, when patients hang up their numbers and enter the waiting area, they generally have plenty of time. In this stage, the first part of the questionnaire was given, and the process took about 8 minutes, including the description of the research purpose and informed consent, EWT, RWT, and personal basic information research. When the patient completed this part of the questionnaire, the first staff recorded the registration time of the patient on the back, wrote a number from 1 to 400 on the back of the questionnaire, and distributed the number to the patient, which was the same as the number of first staff completed on the back of the questionnaire the patient just completed.

In the second stage, when the patient who was about to enter the clinic came to the clinic door, the staff gave the second part of the questionnaire, which had 2 questions in total, (how long he felt he had waited this time, and the other) how satisfied he was with the waiting time for this visit. This part of the questionnaire was completed in 1 minute. After the patients completed this part of the questionnaire, they gave the number, which was obtained in the first stage of the questionnaire, and the second part of the questionnaire to the second staff, which was filled in the number on the back of the questionnaire.

In the third stage, the second staff member recorded the time when the patient entered the clinic in the last line of the questionnaire.

In the data statistics stage, the same number of questionnaires were merged to form a complete questionnaire.

### 2.4. Variables

#### 2.4.1. Demographic variables

The demographic variables included gender, age, hospital history, and education level.

#### 2.4.2. Subjective waiting time

The subjective waiting time variables are EWT, PWT, TWT, and RWT.

#### 2.4.3. Objective waiting time

The objective waiting time variable is AWT, which is defined as the difference between the time of entering the clinic and the time of registration.

#### 2.4.4. Satisfaction level

To assess satisfaction, patients were asked to assign a score between 0 and 100 at random, representing their perception of the visit time, where 0 indicated extreme dissatisfaction and 100 denoted complete satisfaction.

#### 2.4.5. Ethics statement and informed consent

This study was performed in accordance with the Declaration of Helsinki. The study was approved by the Office of Research Ethics of the Eye Hospital of Wenzhou Medical University. Informed consent was obtained from all study participants before completion of the questionnaire.

#### 2.4.6. Statistical analyses

Data analysis was performed using IBM SPSS Statistics 22.0. Multiple linear regression was used to analyze the factors that affect patient satisfaction. At *P* value of <.05, the factor has a significant impact on satisfaction. Considering that waiting times were non-normally distributed, the 2-sample Kolmogorov-Smirnov test and Kruskal–Wallis test were used to analyze the presence of significant differences between basic characters and waiting times. If the *P* value was <.05, a significant difference was considered between them. Descriptive statistical methods were used in this study.

## 3. Results

### 3.1. Characteristics of the participants

A total of 323 questionnaires were collected, including 292 valid questionnaires, with an effective response rate of 90.40%. Among these, 31 questionnaires were rejected because of a lack of logic or key data. For example, someone responded “0” to the question, how long did you think you had to wait for answering?” Another example is that some patients make appointments on-site but fill in the appointment time, which is inconsistent with the actual situation. The patients subjective EWT, TWT, RWT, and other key data were missing and eliminated.

As shown in Table 1, 219 (75%) subjects were women, 155 (53.0%) subjects were between 31 and 40 years old, and 71.8% had a college education or above. Among the 292 patients, 186 (75.6%) made an appointment, and 63.6% had hospital experience. Some basic information of a few patients is missing, but important information, such as waiting times and satisfaction scores, is complete. Therefore, the data were retained during the data analysis.

**Table 1:** Patient demography across survey participants.

Basic information		N (%)
Gender	Male	73 (25)
	Female	219 (75)
Age (yr old)	Under 20 yr old	46 (15.7)
	21–30 yr old	18 (6.1)
	31–40 yr old	155 (53.0)
	41 yr old and above	69 (23.6)
Educational level	High school or below	81 (27.7)
	Diploma or undergraduate	189 (64.7)
	Postgraduate	21 (7.1)
Hospital visit history	Yes	221 (75.6)
	No	61 (20.8)
Appointment	Yes	186 (63.6)
	No	103 (35.2)

### 3.2. Difference of patient waiting time in demography

Significant differences were observed in the waiting times between subjects with and without appointments. The EWT was significantly different between patients with and without appointment (20.0 [10.0, 30.0] vs 30.0 [20.0, 60.0], *P* = .000, *z* = 2.311). Significant differences were also found in PWT (10.0 [10.0, 30.0] vs 30.0 [10.0, 90.0], *P* = .000, z = 2.698), TWT (30.0 [20.0, 60.0] vs 45.0 [30.0, 60.0], *P* = .000, z = 2.355), and RWT (20.0 [10.0, 30.0] vs 30.0 [15.0, 30.0], *P* = .000, z = 2.310). For AWT, a difference was observed (25.0 [13.0, 52.5] vs 147.0 [64.0, 415.0], *P* = .000, z = 5.175). At the same time, the AWT was significantly different at different education levels (64.0 [21.5, 291.0] vs 41.0 [15.25, 88.3] vs 25.0 [15.0, 43.5], *P* = .002, χ^2^ = 12.605). However, significant differences in waiting times were not found for the other factors.

### 3.3. Subjective waiting time has a significant impact on satisfaction, but AWT does not

The factors affecting patient satisfaction were explored using the patient satisfaction score as the dependent variable and personal basic characteristic factors and waiting times as independent variables for linear regression.

Table 2 shows that EWT and LMT have significant impact on satisfaction (*P* = .006, *P* = .043), and they are positively correlated with satisfaction (B *>* *0*, B *>* *0*). For every minute of EWT increase, patient satisfaction increases by 0.08 points. For each minute of TWT increase, patient satisfaction increases by 0.06 points. PWT and RWT also have a significant impact on satisfaction (*P* = .000, *P* = .009), but they were negatively correlated with patient satisfaction (B *<* *0*, B *<* *0*). With a 1 minute increase in PWT, patient satisfaction decreases by 0.17 points. With a 1 minute increase in RWT, patient satisfaction decreases by 0.16 points. However, AWT has no significant effect on satisfaction (*P* = .365). Age, education background, gender, appointment, and hospital visit experience have no significant effect on patient satisfaction (*P* = .105, *P* = .443, *P* = .260, *P* = .352, *P* = .461).

**Table 2:** Analysis of factors affecting satisfaction.

Independent variable	B	Standard error	*t* value	*P* value
The constant	88.362	5.176	17.070	.000†
Age	1.303	0.801	1.628	.105
Educational level	1.085	1.413	.768	.443
Gender	1.804	1.597	1.129	.260
Appointment	1.817	1.949	.932	.352
Hospital visit history	−1.282	1.739	−.738	.461
Perceived waiting time	−.168	.022	−7.759	.000†
Actual waiting time	−.006	.006	−.925	.356
Expected waiting time	.084	.031	2.757	.006†
Reasonable waiting time	−.159	.061	−2.621	.009†
Tolerance waiting time	.058	0.028	2.031	.043*

* *P*<.05

† *P*<.01.

a Dependent variable: satisfaction score; b R square = 0.313, adjusted R square = 0.288, F = 12.381, *P* = .000.

### 3.4. AWT has a significant effect on satisfaction through expectation and perception

The influence of the AWT on satisfaction was not significant. The effect of the gap between the subjective waiting time and AWT on satisfaction was studied. The difference between the EWT and AWT and between the PWT and AWT had a significant effect on satisfaction (both *P* = .000). However, the differences between the RWT and AWT (*P* = .557) and between the TWT and AWT (*P* = .079) had no significant effect on satisfaction.

## 4. Discussion

In addition to the objective waiting time, a patient’s subjective waiting time also plays an important role in the evaluation of medical service quality.^[25]^ The effect of subjective factors, such as EWT, PWT, RWT, and TWT, on patient satisfaction also needs to be determined.

In the present study, patients subjective and objective waiting times that may affect patient satisfaction were investigated immediately in the hospital environment. Almost all studies on patient waiting time and satisfaction have concluded that the longer the AWT, the lower is the patient satisfaction.^[26–29]^ However, some exceptions are observed. In orthopedic rehabilitation clinics, the AWT of patients has no significant impact on satisfaction.^[12]^ AWT does not directly affect satisfaction; however, through the role of EWT and PWT, it has a significant impact on satisfaction, and an answer was obtained from prospect theory. Prospect theory holds that an individual’s evaluation and decision-making depend on the reference point. When the actual situation is above the reference point, the gain is considered, and vice versa.^[17–19]^ In the hospital treatment experience, 2 patients who waited for 1 hour but had different EWT values (e.g., 30 minutes vs 90 minutes) would give different evaluations to the same waiting experience, because when the AWT was longer than the EWT. They felt lost and tended to give negative evaluations. Otherwise, they would have a sense of gain and tend to give positive evaluations. Therefore, although AWT has no significant effect on satisfaction, the effect of AWT on satisfaction is revealed through subjective factors, such as expectations and perceptions. Therefore, reducing patient waiting times and meeting patient expectations can significantly improve outpatient satisfaction.^[30]^

Another finding of this study is consistent with previous research showing that EWT and PWT have an impact on satisfaction.^[13–16]^ In addition to the EWT and PWT, patient RWT and TWT also affect satisfaction. This finding was obtained because almost every patient tolerates some degree of wait time; when the AWT sufficiently exceeds patient norms, they would feel uncomfortable.^[30]^

A significant difference was observed between the subjective and objective waiting times of patients who did and did not make an appointment. This appointment can significantly reduce AWT. Considering the limited medical resources, appointment registration aims to confirm that the patient will see a doctor; however, it is very rare for patients who make an on-site registration to have the opportunity to see a doctor. Therefore, the expectation and perception of waiting time are adjusted accordingly.

## 5. Limitations

The data were obtained from a single hospital. Therefore, we are not sure whether the same results can be obtained in other hospitals. Generally, when a hospital is committed to improving patient satisfaction, it can properly focus on how to adjust the patient’s subjective waiting time, such as adjusting the patient’s EWT through timely information release and optimizing the waiting area environment to reduce patients PWT. These measures can improve patient satisfaction.

In addition, other factors, such as the visiting environment, may affect the patient satisfaction score but cannot be included in the analysis of this study. Previous studies have shown that visiting environment affects patient satisfaction. Similar to all the surveys, an inherent reaction bias was present. The physical discomfort experienced by patients with eye diseases differs from that of other patients. Therefore, the subjects in this study may not represent the experience of the entire patient group.

## 6. Conclusion

This study identified that subjective waiting time directly affects patient satisfaction in outpatients, rather than objective waiting time. When evaluated using the EWT and PWT, the AWT had a significant effect on satisfaction. Therefore, when efforts to reduce AWT have a limited effect on improving patient satisfaction, we can attempt to shift our attention to subjective waiting time. Subjective waiting time is a feasible goal and reliable direction for practical management and scientific research.

## Acknowledgments

The authors thank Neng-Li Wang for valuable comments and suggestions. We also thank the chief editor and the anonymous reviewers for their valuable comments.

## Author contributions

**Conceptualization:** Hui Zhang, Weimin Ma.

**Data curation:** Hui Zhang, Li Wang.

**Formal analysis:** Shufen Zhou, Jingjing Zhu.

**Investigation:** Hui Zhang, Li Wang.

**Methodology:** Shufen Zhou, Jingjing Zhu.

**Project administration:** Weimin Ma, Li Wang.

**Resources:** Jingjing Zhu, Li Wang.

**Supervision:** Weimin Ma, Li Wang.

**Validation:** Hui Zhang, Kaixin Gong.

**Visualization:** Hui Zhang, Kaixin Gong.

**Writing – original draft:** Hui Zhang, Weimin Ma, Li Wang, Kaixin Gong.

**Writing – review & editing:** Hui Zhang, Weimin Ma, Jingjing Zhu.
